# The prevalence of immunoglobulin A nephropathy in the European Union and the impact of the COVID-19 pandemic: an estimation approach utilizing the kidney biopsy frequency

**DOI:** 10.1093/ckj/sfaf068

**Published:** 2025-02-28

**Authors:** Jocelyn Buisker, Sigrid Behr, Nadine Janet Kubesch, Moise E Turkson, Bart Maes, Dmitrij Kollins, Nicholas J A Webb, Jürgen Floege

**Affiliations:** Quantitative Safety and Epidemiology, Novartis Pharma AG, Basel, Switzerland; Quantitative Safety and Epidemiology, Novartis Pharma AG, Basel, Switzerland; Life Science-Intuitive Operation and Automation, Cognizant Technology Solutions, Barcelona, Spain; Life Science-Intuitive Operation and Automation, Cognizant Technology Solutions, London, UK; Department of Nephrology, AZ Delta, Roeselare, Belgium; Clinical Development, Novartis Pharma AG, Basel, Switzerland; Clinical Development, Novartis Pharma AG, Basel, Switzerland; Division of Nephrology and Rheumatology, RWTH Aachen University Hospital, Aachen, Germany

**Keywords:** epidemiology, glomerulonephritis, IgA nephropathy, kidney biopsy, systematic review

## Abstract

**Background:**

Immunoglobulin A nephropathy (IgAN) is the most common type of primary glomerulonephritis, requiring a kidney biopsy for diagnosis. This study aimed to estimate the prevalence of primary IgAN within the European Union (EU) and investigate the potential impact of the coronavirus disease 2019 (COVID-19) pandemic on kidney biopsy rates and IgAN diagnosis frequency.

**Methods:**

We conducted four comprehensive literature searches to identify data on the IgAN prevalence and incidence, native kidney biopsy rates and COVID-19 impact on these metrics. We calculated country-specific prevalence estimates based on a combination of published and modeled data, incorporating biopsy frequency and the Healthcare Access and Quality Index (HAQI). The EU IgAN prevalence was derived from country-specific prevalence estimates weighted by population size.

**Results:**

The estimated prevalence of IgAN in the EU was 4.31 per 10 000 population, with large geographic variation among countries, ranging from 0.16 to 14.4 per 10 000 population. A strong correlation was observed between the IgAN incidence and biopsy rate (R^2^ = 0.96). Countries with a higher HAQI mostly exhibited higher biopsy rates and IgAN incidences. The COVID-19 pandemic resulted in a notable decrease in kidney biopsy rates for most European countries with available information in 2020 compared with both pre- and post-pandemic periods. However, the long-term implications of this reduction on biopsy rates and subsequent IgAN incidence remain to be determined.

**Conclusion:**

Our findings confirm the rarity of IgAN, albeit the most common type of glomerulonephritis. They underscore a robust correlation between biopsy rates and IgAN incidence, influenced by healthcare access and quality. The COVID-19 pandemic's temporary suppression of biopsy rates in 2020 suggests potential delays in IgAN diagnosis, warranting further investigation into the long-term effects.

KEY LEARNING POINTS **What was known:**

- Immunoglobulin A nephropathy (IgAN) is a rare disease, and its occurrence varies significantly across different regions and healthcare systems, notable even within the borders of a single country.

**This study adds:**

- The prevalence of IgAN in the European Union (EU) was 4.31 per 10 000 population estimated based on a combination of published and modeled data, incorporating biopsy frequency and the Healthcare Access and Quality Index.
- We found a strong correlation between the native kidney biopsy rate and the IgAN incidence across European countries that persisted throughout time and highlighted the role of healthcare access and quality related factors as drivers of the kidney biopsy rate and IgAN incidence.
- In addition, we provided published and modeled IgAN incidence and prevalence estimates for all EU countries, including those without previously published information on the IgAN frequency.

**Potential impact:**

- Our analysis clearly confirmed that IgAN is a rare disease in the EU. The COVID-19 pandemic led to a temporary decrease in kidney biopsy rates in some countries, resulting in potential delays in IgAN diagnosis.
- In the future, biopsy rates and the number of IgAN cases might increase, given (i) the approval of IgAN specific drugs, which may alleviate the historical nihilism, and (ii) the realization that urinary abnormalities may carry a significant lifetime risk of kidney failure.

## INTRODUCTION

Immunoglobulin A nephropathy (IgAN) is the most common form of primary glomerulonephritis and represents a significant cause of chronic kidney disease and kidney failure worldwide. The diagnosis of IgAN requires a kidney biopsy to determine the presence of IgA deposits. IgAN can occur as primary, or secondary in association with other conditions [1]. The pooled IgAN prevalence across 10 European countries has previously been reported as 2.53 per 10 000 population [2]. However, there is a significant variation in disease frequency across different regions [1, 3] and several studies have identified a rising number of IgAN cases over time, contributing to the challenge of estimating its true prevalence [4–9]. IgAN epidemiology estimates cannot be easily generalized from one region to broader or different populations. The variation in IgAN frequency is notable even within the borders of a single country [10, 11], making it difficult to assess how common IgAN is on a larger scale, such as within the European Union (EU). One plausible factor behind this variation might be the extent to which kidney biopsies are performed; higher rates of biopsies may lead to the detection of more IgAN cases [11].

Another factor potentially influencing the number of IgAN diagnoses is the coronavirus disease 2019 (COVID-19) pandemic. It largely impacted healthcare access across the globe, prompting the question of whether it influenced kidney biopsy rates to such an extent that it has had long-lasting effects on the apparent prevalence of IgAN, potentially lasting until today.

Our aim was to estimate the prevalence of primary IgAN in the EU utilizing the native kidney biopsy frequency to complement the scarce country-specific disease epidemiology data. In addition, we investigated potential effects of the COVID-19 pandemic on the kidney biopsy and IgAN frequency.

## MATERIALS AND METHODS

### Literature review

Four comprehensive literature reviews were conducted in Ovid MEDLINE^®^ to identify publications of population-based studies from Europe (51 continental and transcontinental countries) including well-defined national or regional populations of all ages or adults published until 15 March 2024. Publications in English language reporting information on, or sufficient data to calculate (i) the prevalence or incidence of primary IgAN diagnosed by native kidney biopsy, (ii) the native kidney biopsy rate, (iii) the COVID-19 pandemic impact on the IgAN incidence, or (iv) the pandemic impact on the native kidney biopsy frequency were included. We excluded studies not fulfilling the inclusion criteria, on IgA vasculitis, and low-quality studies as judged by two epidemiologists, i.e. inappropriate definition of incidence or prevalence, study population, or containing a high risk of bias (Supplementary data, S1.1).

As IgAN is a rare disease, thus the incidence proportion, i.e. the number of new cases in a specified time period divided by the number of persons at the start of the specified time period, and the incidence rate, i.e. the number of new cases in a specified time period divided by the total person-time at risk, were treated as equal. While we acknowledge that most estimates of native kidney biopsy frequency were reported as cumulative incidences rather than actual rates, we refer to them as “(kidney) biopsy rates” throughout the article. Throughout the article, we use the annual incidence or biopsy rate without explicitly stating “annual,” unless further specified.

### Statistical analysis

Quantitative analyses were performed using SAS (Release 3.81, SAS Institute Inc., Cary, NC, USA). All presented numbers were rounded to two decimal places.

#### Data preparation

Data relevant for the prevalence analysis were extracted from publications fulfilling the inclusion and exclusion criteria of the literature reviews (i) and (ii).

The IgAN point prevalence was defined as the proportion of patients with primary IgAN among a well-defined population at a specified point in time. The IgAN incidence was determined as the number of patients newly diagnosed with primary IgAN in 1 year divided by the number of patients at risk in the population during the same year. The annual biopsy rate was similarly defined by the number of patients undergoing a native kidney biopsy for any reason in 1 year divided by the population at risk during the same year. If incidence or biopsy rates were not directly reported, they were calculated based on the number of cases, number of years in the study period and the population at risk. The population at risk was preferably used as reported in the respective publication, or else from official statistics such as Eurostat (https://ec.europa.eu/eurostat), World Bank (https://www.worldbank.org/en/home) and country-specific statistics (Supplementry data, S2).

#### Prevalence calculation

Country-specific IgAN prevalence estimates across the 27 EU countries were retrieved, either used as reported in the underlying studies, or calculated by multiplying the IgAN incidence by the disease duration. The disease duration was assumed as 30 years based on the median time from diagnosis to kidney failure [12] considering the time of kidney failure as the end of primary IgAN diagnosed through native kidney biopsy.

If several high-quality prevalence or incidence estimates were available for one country, the most recent estimate was used. Since we expected few publications reporting the IgAN prevalence, we decided to handle potential inconsistencies in the underlying prevalence estimation methods on a case-by-case basis as judged by epidemiologists. If no country-specific IgAN prevalence or incidence estimates were available, the IgAN incidence was estimated through a linear regression analysis by modeling the annual IgAN incidence as a linear function of the annual biopsy rate based on published data from European countries and regions. The incidence was estimated by multiplying the country-specific biopsy rate by the regression slope. If there was also no available relevant biopsy rate, the Healthcare Access and Quality Index (HAQI) as reported in the Global Burden of Disease study was utilized to make a reasonable assumption about the biopsy rate [13, 14]. The HAQI was divided into four categories (low, medium-low, medium-high, high) based on the range of HAQI across European countries. For countries without any relevant information, the median biopsy rate of all available biopsy rates from European neighbor countries within the same HAQI category was assumed instead. In case of no neighbors within the same HAQI category with available biopsy rate estimates, the median biopsy rate of all countries within the respective HAQI category was assumed. The country-specific prevalence was then estimated as described before (for detailed methods see Supplementary data, S1.2).

**Figure 1:**
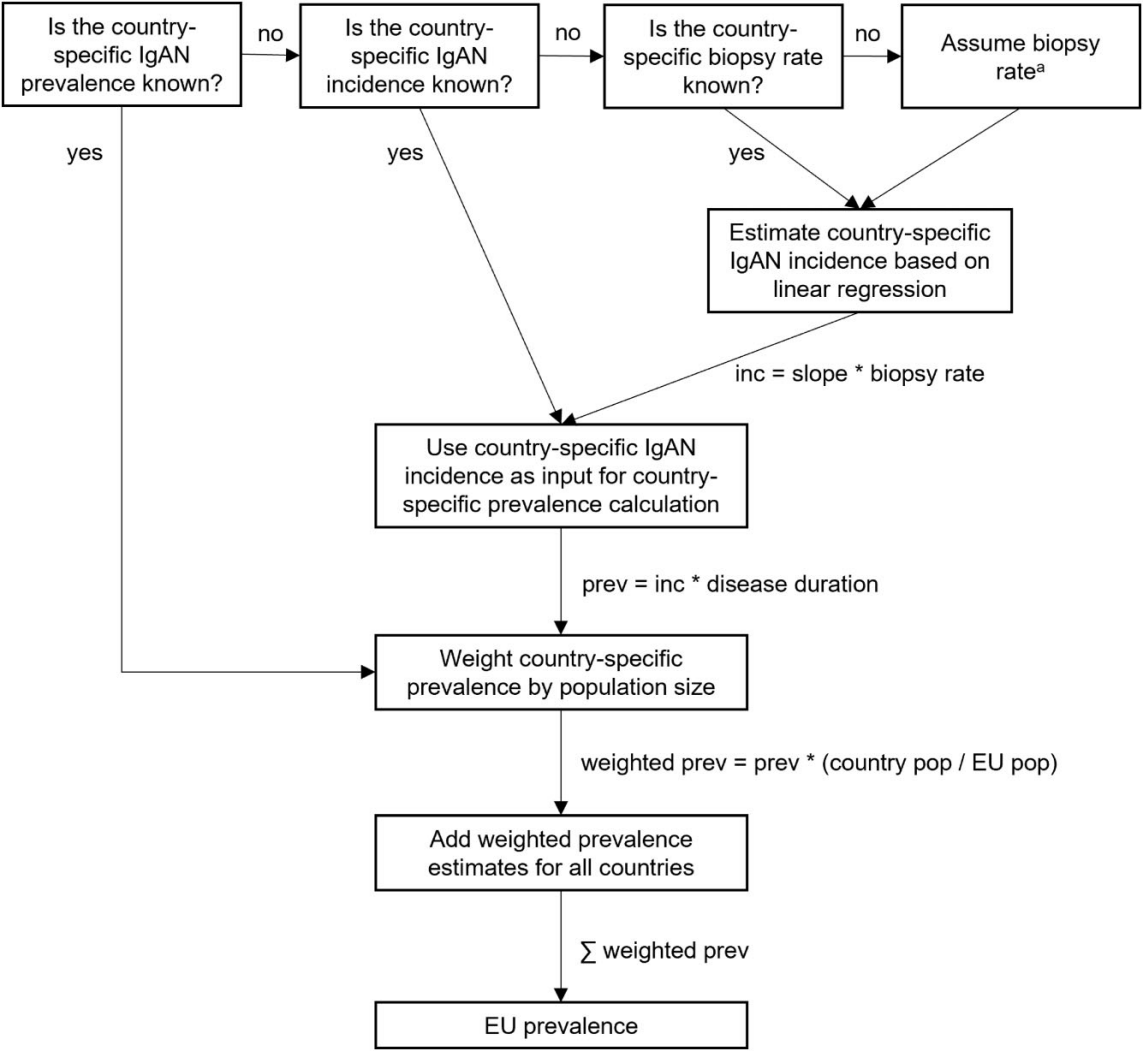
Graphical depiction of EU IgAN prevalence estimation leveraging the linear relationship between biopsy rate and IgAN incidence. ^a^The assumed biopsy rate was the median of all reported biopsy rates of direct European neighbor countries within the same HAQI category. Abbreviations: inc, incidence; pop, population; prev, prevalence.

Subsequently, the IgAN prevalence in the EU was estimated by the sum of country-specific prevalence estimates weighted by the country-specific population size relative to the EU population size using population counts of 2023. A graphical depiction of the prevalence estimation process is shown in Fig. 1.

Robustness of results regarding the assumptions for the disease duration and biopsy rates for countries without relevant information was evaluated in four sensitivity analyses (Supplementary data, Table S3.2).

### Assessment of COVID-19 impact

We identified publications of annual data on the biopsy or IgAN frequency covering the pre-pandemic period (before 2020) throughout the end of 2020 or longer in literature reviews (iii) and (iv) complemented by a grey literature search which we qualitatively analyzed and discussed.

## RESULTS

### IgAN incidence

The literature reviews (i) and (ii) identified 32 relevant publications from 22 European countries reporting on the incidence of IgAN and/or the native kidney biopsy rate in European countries. The PRISMA (Preferred Reporting Items for Systematic Reviews and Meta-Analyses) diagram is provided in Supplementary data, Fig. S3.1. No relevant article directly measuring the country-specific IgAN prevalence in high quality could be identified.

**Figure 2:**
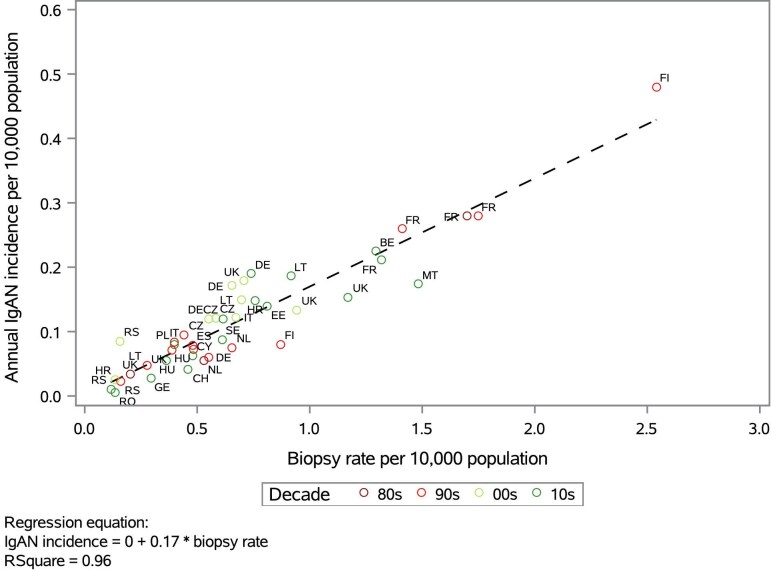
Linear regression of annual native kidney biopsy rate and annual IgAN incidence with datapoints marked by country and decade of the mid-point of the study period interval. Abbreviations: BE, Belgium; CH, Switzerland; CY, Cyprus; CZ, Czech Republic; DE, Germany; DK, Denmark; EE, Estonia; ES, Spain; FI, Finland; FR, France; GE, Georgia; HR, Croatia; HU, Hungary; IE, Ireland; IT, Italy; LT, Lithuania; MT, Malta; NL, Netherlands; PL, Poland; RO, Romania; RS, Serbia; SE, Sweden; UK, United Kingdom; 00s, 2000s; 10s, 2010s; 80s, 1980s; 90s, 1990s.

The relationship of the IgAN incidence and biopsy rate based on reported data from underlying studies was inspected graphically for potential patterns by country and study period. There was no visible cluster of IgAN incidence and biopsy rate estimates by decade (Fig. 2). We observed a clear linear relationship between the IgAN incidence and biopsy rate (IgAN incidence = 0.17 * biopsy rate; R^2 ^= 0.96) (Fig. 2). The biopsy rate estimates in Europe ranged from 0.12 to 2.54, and the incidence estimates from 0.01 to 0.48 per 10 000 population. The input data for the regression are presented in Table 1.

**Table 1:** Input data for the linear regression of IgAN incidence and biopsy rate identified from scientific publications.

Country	Study period(s)	Annual biopsy rate per 10 000 population	Annual IgAN incidence per 10 000 population	Reference
Belgium (BE)	2017–2019	1.30	0.23	[29]
Croatia (HR)	1996–2011	0.14^a^,^b^	0.03^a^,^b^	[30]
Croatia (HR)	2019	0.76^a^,^b^	0.15^a^,^b^	[31]
Cyprus (CY)	2006–2015	0.48	0.06	[32]
Czech Republic (CZ)	1994	0.44	0.09^a^,^b^	[4]
	2001	0.58	0.12^a^,^b^	
	2011	0.62	0.12^a^,^b^	
Estonia (EE)	2010	0.81	0.14	[24, 33]
Finland (FI)	1980–2000	University hospital: 2.54	University hospital: 0.48	[24]
		Central hospital: 0.87	Central hospital: 0.08	
France (FR)	2015–2017	1.32	0.21^a^	[34]
France (FR)	1976–1985	1.70	0.28	[35]
	1986–1995	1.75	0.28	
	1996–2002	1.41	0.26	
Georgia (GE)	2011–2020	0.29^a^	0.03^a^	[15]
Germany (DE)	2003–2008	0.66	0.17	[36]
Germany (DE)	1990–1997	0.55	0.06	[6]
	1998–2005	0.55	0.12	
	2006–2013	0.74	0.19	
Hungary (HU)	2006–2020	0.36	0.05^a^	[16]
Hungary (HU)	1990–2002	0.48^a^,^b^	0.07^a^,^b^	[37]
Italy (IT)	1993	0.40^a^,^b^	0.08	[38]
Italy (IT)	1998–2010	0.67^a^	0.12	[39]
Lithuania (LT)	1994–1999	0.28^a^	0.05^a^	[9]
	2000–2006	0.70^a^	0.15^a^	
	2007–2012	0.92^a^	0.19^a^	
Malta (MT)	2014–2018	1.48^a^,^b^	0.17^a^,^b^	[40]
Netherlands (NL)	1978–1990	0.53^a^,^b^	0.06^a^,^b^	[5]
	1991–2003	0.65^a^,^b^	0.07^a^,^b^	
Poland (PL)	2014	0.40	0.08^a^	[10]
Romania (RO)^c^	2011–2019	0.14	0.01^a^	[41]
Serbia (RS)	2008–2014	0.12	0.01^a^	[23]
Serbia (RS)	2000–2006	0.16	0.09	[42]
Serbia (RS)	1986–2006	0.16	0.02^a^	[43]
Spain (ES)	1994–1999	0.48	0.08	[44]
Sweden (SE)	2006–2013	0.61^a^	0.09^a^	[45]
Switzerland (CH)	2007–2016	0.46^a^	0.04	[46]
United Kingdom (UK)	1976–1985	0.20	0.03	[7]
	1986–1995	0.39	0.07	
	1996–2005	0.71	0.18	
United Kingdom (UK)	2014–2021	1.17	0.15^a^	[47]
United Kingdom (UK)	2000–2010	0.94	0.13^a^	[48]
Europe [median (IQR)]		0.60 (0.40–0.87)	0.09 (0.06–0.17)	

All numbers were rounded to two decimal places.

a Calculated by authors based on published data on the number of IgAN cases and population denominator.

b Denominator for calculation from official population statistics.

c Estimates were based on mesangial proliferative glomerulonephritis only but was included as an exception as there is no IgAN-specific data for Romania.

IQR, interquartile range.

For EU countries without reported IgAN incidence and biopsy rate, the median of all reported biopsy rates from European neighbor countries within the same HAQI category was used. The IgAN incidence was then calculated through the linear regression model (Supplementary data, Table S3.1). Reported and calculated IgAN incidence estimates in EU countries ranged between 0.01 and 0.48 per 10 000 population (Table 2).

**Table 2:** Calculation of IgAN prevalence in EU countries.

EU country	Annual incidence per 10 000 population	Country prevalence per 10 000 population	Population in 2023 (Eurostat 2024)	Reference
Austria	0.09	2.80	9 104 772	Calculated^a^
Belgium	0.23	6.75	11 742 796	[29]
Bulgaria	0.02	0.69	6 447 710	Calculated^a^
Croatia	0.15	4.45	3 850 894	[31]
Cyprus	0.06	1.89	920 701	[32]
Czech Republic	0.12	3.57	10 827 529	[4]
Denmark	0.11	3.21	5 932 654	[49]
Estonia	0.14	4.20	1 365 884	[33]
Finland	0.48	14.40	5 563 970	[24]
France	0.26	7.80	68 172 977	[35]
Germany	0.19	5.70	84 358 845	[6]
Greece	0.11	3.43	10 413 982	Calculated^a^
Hungary	0.05	1.64	9 599 744	[16]
Ireland	0.12	3.61	5 271 395	Calculated^a^
Italy	0.12	3.66	58 997 201	[39]
Latvia	0.12	3.55	1 883 008	Calculated^a^
Lithuania	0.19	5.59	2 857 279	[9]
Luxembourg	0.22	6.59	660 809	Calculated^a^
Malta	0.17	5.23	542 051	[40]
Netherlands	0.07	2.25	17 811 291	[5]
Poland	0.08	2.40	36 753 736	[10]
Portugal	0.08	2.44	10 467 366	Calculated^a^
Romania^b^	0.01	0.16	19 054 548	[41]
Slovakia	0.08	2.35	5 428 792	Calculated^a^
Slovenia	0.09	2.73	2 116 972	Calculated^a^
Spain	0.08	2.40	48 085 361	[44]
Sweden	0.13	3.88	10 521 556	[50]
EU [median (IQR)]	0.12 (0.08–0.17)	3.55 (2.40–5.23)		

All numbers were rounded to two decimal places.

a Modeled from linear regression between biopsy rate and IgAN incidence using the median biopsy rate of European neighbor countries with available data within the same HAQI category as input data.

b Estimates were based on mesangial proliferative glomerulonephritis only but was included as an exception as there is no IgAN-specific data for Romania.

IQR, interquartile range.

### IgAN prevalence

**Figure 3:**
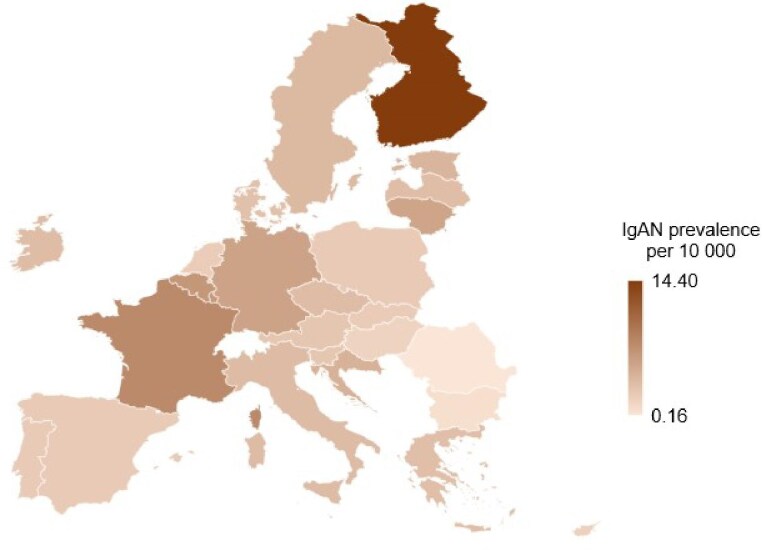
The estimated prevalence of IgAN across EU countries (reported per 10 000 population).

The IgAN prevalence in the EU was estimated as 4.31 per 10 000 population based on published data from 18 EU countries and calculated data for the remaining nine countries. The country-specific estimates ranged from 0.16 in Romania to 14.4 per 10 000 in Finland (Table 2, Fig. 3).

### Impact of COVID-19 pandemic

**Figure 4:**
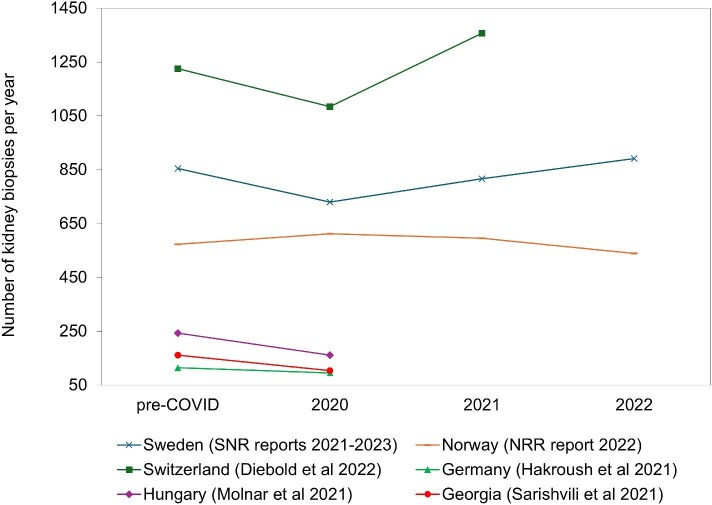
The frequency of kidney biopsies before, and throughout the COVID-19 pandemic in Europe as reported by scientific publications and registry reports.

Most available publications on the biopsy rate or IgAN incidence before, during and after the COVID-19 pandemic in Europe reported a reduced biopsy frequency in 2020 [15–21]. Only Norway reported a consistent biopsy frequency from pre-pandemic years throughout the pandemic until 2022 (Fig. 4) [22]. The available evidence on the IgAN incidence in European countries in 2020 was sparse, but suggested a reduced number of IgAN diagnoses in 2020 compared with the years before and after [20, 21].

## DISCUSSION

### IgAN epidemiology and biopsy rate

We estimated the IgAN prevalence in the EU as 4.31 per 10 000 population based on published data and the strong linear relationship between the biopsy rate and IgAN incidence while considering geographic differences in healthcare access and quality.

Biopsy rates highly depend on the healthcare policy in the respective region. There seemed to be a general pattern of countries with a higher HAQI reporting a higher biopsy rate and IgAN incidence than countries with a lower HAQI. Furthermore, countries in the eastern parts of Europe showed a lower HAQI than countries in the western parts (Supplementary data, Figs S3.2 and S3.3) which was consistent with the observation that the highest reported biopsy rates and IgAN incidences were often from western and the lower from eastern countries (Fig. 2). The lowest biopsy rate was reported in Serbia [23]. Serbia was assigned a medium-high HAQI while numerically being in the lower half of those reported for European countries (Supplementary data, Fig. S3.2) which might explain the relatively low biopsy rate. The highest biopsy rate was observed in a Finnish university hospital; however, it was not representative for the whole country since the same study showed a substantially lower biopsy rate in other Finnish central hospitals [24]. A number of studies suggested that biopsy rates could differ to a large extent even under the same healthcare system [10, 11, 24], indicating that availability of nephrology departments and the presence of “centers of excellence,” institutional policies and extents of the nephrologist's network might be contributing factors.

Results from our study did not reveal clear temporal patterns on a European level (Fig. 2). This observation does not contradict the existence of time trends in smaller geographies or on national levels [4–9] but highlighted that the role of the kidney biopsy rate as a strong driver of the IgAN incidence has been consistent across time. In the future, biopsy rates and the number of IgAN cases might increase, given the approval of IgAN specific drugs, which may alleviate the historical nihilism, and the realization that urinary abnormalities may carry a significant lifetime risk of kidney failure.

Another study reviewing the IgAN prevalence in Europe reported a lower prevalence, i.e. 2.53 per 10 000 population, estimated based on scientific publications from 10 European countries [2]. While the geographic scope of the prevalence estimation was slightly different, the main driver for the differences in prevalence were design aspects. The authors included only registry-based studies with national coverage, while this analysis also included regional data, and applied a modelling approach for the countries without reported IgAN incidence or kidney biopsy rates.

### COVID-19 impact on kidney biopsy rate and IgAN incidence

The year 2020 marked the beginning of the COVID-19 pandemic in Europe which had large impacts on healthcare across the continent. Several studies across Europe reported a decreasing native kidney biopsy frequency in 2020 [15–21]. Primarily, patients with less severe symptoms [16, 20, 21], younger patients, and women seemed to be affected [17]. Potential reasons were reported as the switch of nephrology departments to urgent care reducing the number of biopsies, patients avoiding hospital visits [16], limited access to primary care, and consequent reduction in referrals to secondary care during the pandemic [25].

A national study from Switzerland noted a reduced IgAN incidence during the first infection wave and suggested that IgAN patients often had less severe or no symptoms which increased the chance to miss IgAN cases with reduced routine medical care [21]. A single center study from the UK also observed a lower IgAN incidence in 2020 compared with pre-pandemic years and 2021, likely explained by limited healthcare access [25].

The total number of biopsies performed in a center in Germany was non-significantly reduced in 2020 compared with 2019 which was explained by a reduced biopsy rate in March and April 2020, and a compensatory rate in the subsequent months. The decrease in the number of biopsies was observed in the normal medical ward while intensive and emergency care were not affected [20].

The Norway Renal Registry did not observe a decrease in the number of biopsies in 2020 [22]. Norway was among the countries least affected by the first severe acute respiratory syndrome coronavirus 2 (SARS-CoV-2) variants, and managed to control the situation in the first wave rather quickly. The country was therefore able to lift restrictions early on in 2020. Additionally, hospital resources apart from emergency care were not much affected, and kidney biopsies were handled on a day-post which might explain that the frequency of kidney biopsies did not decrease in 2020 [26, 27].

While most of the published evidence pointed towards a decreased biopsy rate across Europe during the COVID-19 pandemic, it is unclear whether it had long-lasting effects on the kidney biopsy rate or IgAN incidence that would affect today's IgAN prevalence. There have been hints towards an increased biopsy rate following the high phase of the pandemic [18, 20] but it has not been shown on a European scale. More recent annual data are needed to assess how the biopsy rate and IgAN incidence developed after 2020 to make conclusions on whether the COVID-19 pandemic affects the current IgAN prevalence.

### Strengths and limitations

We included well-defined regional data for countries without national data, potentially leading to an overestimation of the diagnosed IgAN prevalence. Regions with special interest in IgAN or in general kidney diseases, for example due to the existence of a “center of excellence,” might conduct more kidney biopsies and be more likely to publish on this topic leading to an overestimation of the IgAN frequency when extrapolating the data to the whole country. However, by including regional studies we could increase the robustness of the linear regression, and thus, of the prevalence estimation. Our aim was to get an as complete as possible picture of the IgAN prevalence in the EU, and we believed that the assumption of a regional prevalence for a given country was more appropriate than not considering that country in the estimation. Additionally, we chose to model the IgAN frequency for countries without relevant information based on the biopsy rates from neighboring countries with a similar HAQI to improve the validity of extrapolating biopsy rates from one country to another. The assumptions made for extrapolation, and more so for estimating the prevalence based on the annual incidence rate, limit the accuracy of the prevalence, as can be seen from the sensitivity analyses (Supplementary data, Table S3.2). However, the range of prevalence estimates obtained from the sensitivity analyses is substantially smaller than the range of country-specific prevalence estimates. Therefore, considering the scarcity of relevant data and in absence of an European effort to collect data with a common standard, we believe that this work contributes to a more complete description of the IgAN frequency across the EU.

We estimated the diagnosed IgAN prevalence which may not include all truly existing IgAN cases. It is difficult to account for undiagnosed cases due to several reasons. First, IgAN is diagnosed through kidney biopsy. Kidney biopsy is an invasive method potentially leading to complications. Thus, physicians might not biopsy all suspected patients, in particular in the historical context of little available treatment beyond supportive care, and especially when they have a mild disease, or belong to a vulnerable population such as the pediatric population. Second, in almost all European countries there is no broad screening for potential IgAN symptoms like urine abnormalities in the general population, and even if urine abnormalities are detected, general practitioners might ignore the findings or refer patients to urology rather than nephrology. Additionally, IgAN may exist subclinically and can therefore stay undetected for prolonged periods without a clear need for treatment [1]. Finally, not all kidney biopsies may be examined by immunohistology in some countries due to financial restraints, leading to the misclassification of IgAN as another entity. While we acknowledge the efforts and challenges of collecting IgAN data, the listed limitations highlight the need for a common European standard moving away from siloed approaches which result in a high variability of published estimates and interpretation difficulties.

We conducted every step of this project based on a pre-specified and agreed analysis plan, and all steps, i.e. data extraction and preparation, quantitative and qualitative analysis, as well as results presentation were performed by two researchers to ensure a high quality. However, the full-text screening during the literature search was not done by two independent researchers. As the literature search was complemented by a grey literature search, we do not believe that we missed important publications which might lead to a substantial impact on the results.

### Conclusion

Our analysis clearly confirmed that IgAN is a rare disease in the EU [28]. We found a strong correlation between the native kidney biopsy rate and the IgAN incidence across European countries that persisted throughout time and highlighted the role of factors related to healthcare access and quality as drivers of the kidney biopsy rate and IgAN incidence. In addition, we provided IgAN incidence and prevalence estimates for countries without previously published information on the IgAN frequency. While the COVID-19 pandemic led to a temporary decrease in kidney biopsy rates in some countries, resulting in potential delays in IgAN diagnosis, the long-term effects on the IgAN frequency remain to be fully understood.

## Supplementary Material

sfaf068_Supplemental_Files

## Data Availability

The data to support the findings are available in Supplement 2.

## References

[1] Du Y, Cheng T, Liu C et al. IgA nephropathy: current understanding and perspectives on pathogenesis and targeted treatment. Diagnostics 2023;13:303. 10.3390/diagnostics13020303 36673113 PMC9857562

[2] Willey CJ, Coppo R, Schaefer F et al. The incidence and prevalence of IgA nephropathy in Europe. Nephrol Dial Transplant 2023;38:2340–9. 10.1093/ndt/gfad082 37156519 PMC10539204

[3] Schena FP, Nistor I. Epidemiology of IgA nephropathy: a global perspective. Semin Nephrol 2018;38:435–42. 10.1016/j.semnephrol.2018.05.013 30177015

[4] Maixnerova D, Jancova E, Skibova J et al. Nationwide biopsy survey of renal diseases in the Czech Republic during the years 1994–2011. J Nephrol 2015;28:39–49. 10.1007/s40620-014-0090-z 24756969

[5] Van Paassen P, Van Breda Vriesman PJC, Van Rie H et al. Signs and symptoms of thin basement membrane nephropathy: a prospective regional study on primary glomerular disease—The Limburg Renal Registry. Kidney Int 2004;66:909–13. 15327380 10.1111/j.1523-1755.2004.00835.x

[6] Zink CM, Ernst S, Riehl J et al. Trends of renal diseases in Germany: review of a regional renal biopsy database from 1990 to 2013. Clin Kidney J 2019;12:795–800. 10.1093/ckj/sfz023 31808446 PMC6885677

[7] Hanko JB, Mullan RN, O'Rourke DM et al. The changing pattern of adult primary glomerular disease. Nephrol Dial Transplant 2009;24:3050–4. 10.1093/ndt/gfp254 19487734

[8] Stratta P, Segoloni GP, Canavese C et al. Incidence of biopsy-proven primary glomerulonephritis in an Italian province. Am J Kidney Dis 1996;27:631–9. 10.1016/S0272-6386(96)90096-7 8629621

[9] Brazdziute E, Miglinas M, Gruodyte E et al. Nationwide renal biopsy data in Lithuania 1994–2012. Int Urol Nephrol 2015;47:655–62. 10.1007/s11255-015-0927-y 25686739

[10] Perkowska-Ptasinska A, Bartczak A, Wagrowska-Danilewicz M et al. Clinicopathologic correlations of renal pathology in the adult population of Poland. Nephrol Dial Transplant 2017;32:ii209–18. 10.1093/ndt/gfw365 28339709

[11] McQuarrie EP, MacKinnon B, Young B et al. Centre variation in incidence, indication and diagnosis of adult native renal biopsy in Scotland. Nephrol Dial Transplant 2009;24:1524–8. 10.1093/ndt/gfn677 19074409

[12] Jarrick S, Lundberg S, Welander A et al. Mortality in IgA nephropathy: a nationwide population-based cohort study. J Am Soc Nephrol 2019;30:866–76. 10.1681/ASN.2018101017 30971457 PMC6493992

[13] Institute for Health Metrics and Evaluation (IHME) at the University of Washington . (18 November 2024, date last accessed). Used with permission. https://www.healthdata.org/research-analysis/gbd

[14] Haakenstad A, Yearwood JA, Fullman N et al. Assessing performance of the Healthcare Access and Quality Index, overall and by select age groups, for 204 countries and territories, 1990–2019: a systematic analysis from the Global Burden of Disease Study 2019. Lancet Glob Health 2022;10:e1715–43. 10.1016/S2214-109X(22)00429-6 36209761 PMC9666426

[15] Sarishvili N, Tchokhonelidze I, Tevdoradze T et al. MO312 Main trends and outcomes of kidney disease in Georgia: the first review of kidney biopsy database from 2011 to 2020. Nephrol Dial Transplant 2021;36:i230. 10.1093/ndt/gfab104.0070

[16] Molnár A, Thomas MJ, Fintha A et al. Kidney biopsy-based epidemiologic analysis shows growing biopsy rate among the elderly. Sci Rep 2021;11:24479. 10.1038/s41598-021-04274-9 PMC8716536 34966177

[17] SRR . Svenskt Njurregister ÅRsrapport 2021 [Internet]. Jönköping: 2021; Available from: https://www.medscinet.net/snr/arsrapporter.aspx [19 July 2024, date last accessed].

[18] SRR . Svenskt Njurregister ÅRsrapport 2023 [Internet]. Jönköping: 2023; Available from: https://www.medscinet.net/snr/arsrapporter.aspx [19 July 2024, date last accessed].

[19] SRR . Svenskt Njurregister ÅRsrapport 2022 [Internet]. Jönköping: 2022; Available from: https://www.medscinet.net/snr/arsrapporter.aspx [19 July 2024, date last accessed].

[20] Hakroush S, Tampe D, Korsten P et al. Impact of the COVID-19 pandemic on kidney diseases requiring renal biopsy: a single center observational study. Front Physiol 2021;12. 10.3389/fphys.2021.649336 PMC8297651 34305628

[21] Diebold M, Locher E, Boide P et al. Incidence of new onset glomerulonephritis after SARS-CoV-2 mRNA vaccination is not increased. Kidney Int 2022;102:1409–19. 10.1016/j.kint.2022.08.021 36096267 PMC9462927

[22] NRR . The Norwegian Renal Registry (Norsk Nyreregister)—Annual Report [Internet]. Oslo: 2022; Available from: https://nephro.no/nnr/AARSRAPPORT_NNR_2022.pdf [13 March 2025, date last accessed].

[23] Brkovic V, Milinkovic M, Kravljaca M et al. Does the pathohistological pattern of renal biopsy change during time? Pathol Res Pract 2018;214:1632–7. 10.1016/j.prp.2018.07.027 30139556

[24] Wirta O, Mustonen J, Helin H et al. Incidence of biopsy-proven glomerulonephritis. Nephrol Dial Transplant 2008;23:193–200. 10.1093/ndt/gfm564 17720989

[25] Boothroyd PG, Chu K, Sinha SC et al. TH-PO906: Long COVID effect of the COVID-19 pandemic on incidence of glomerular disease: a single centre report. J Am Soc Nephrol 2022;33:2022. 10.1681/ASN.20223311S1307d

[26] Christensen T, Lægreid P. Balancing governance capacity and legitimacy: how the Norwegian government handled the COVID-19 crisis as a high performer. Public Adm Rev 2020;80:774–9. 10.1111/puar.13241 32836445 PMC7280699

[27] Hovd M, Åsberg A, Munthe LA et al. Humoral vaccine response and breakthrough infections in kidney transplant recipients during the COVID-19 pandemic: a nationwide cohort study. eClinicalMedicine 2023;60:102035. 10.1016/j.eclinm.2023.102035 37362086 PMC10242148

[28] European Commission . Rare Diseases [Internet]. Brussels: 2024; Available from: https://research-and-innovation.ec.europa.eu/research-area/health/rare-diseases_en [13 September 2024, date last accessed].

[29] Laurens W, Deleersnijder D, Dendooven A et al. Epidemiology of native kidney disease in Flanders: results from the FCGG kidney biopsy registry. Clin Kidney J 2022;15:1361–72. 10.1093/ckj/sfac033 35756729 PMC9217646

[30] Horvatic I, Tisljar M, Bulimbasic S et al. Epidemiologic data of adult native biopsy-proven renal diseases in Croatia. Int Urol Nephrol 2013;45:1577–87. 10.1007/s11255-013-0397-z 23456817

[31] Laganović M, Gellineo L, Bulimbašić S et al. Report of the croatian registry of native kidney biopsies for year 2019. Acta Clinica Croatica 2021;60:173–80.

[32] Oygar DD, Neild GH. Reporting renal biopsies from cyprus: a systematic approach. J Nephropathol 2017;6:231–9. 10.15171/jnp.2017.38 28975106 PMC5607988

[33] Riispere Ž, Ots-Rosenberg M. Occurrence of kidney diseases and patterns of glomerular disease based on a 10-year kidney biopsy material: a retrospective single-centre analysis in Estonia. Scand J Urol Nephrol 2012;46:389–94. 10.3109/00365599.2012.693133 22725262

[34] Asgarali E, Gayon J, Viallet N et al. Kidney biopsy in Reunion Island between 2015 and 2017, an epidemiological study. Nephrol Ther 2021;17:512–9. 34548266 10.1016/j.nephro.2021.06.006

[35] Simon P, Ramee MP, Boulahrouz R et al. Epidemiologic data of primary glomerular diseases in western France. Kidney Int 2004;66:905–8. 15327379 10.1111/j.1523-1755.2004.00834.x

[36] Braun N, Schweisfurth A, Lohöfener C et al. Epidemiology of glomerulonephritis in Northern Germany. Int Urol Nephrol 2011;43:1117–26. 10.1007/s11255-011-9955-4 21505754

[37] Sipiczki T, Ondrik Z, Abraham G et al. The incidence of renal diseases as diagnosed by biopsy in Hungary. Orv Hetil 2004;145:1373–9. 15384747

[38] Schena FP . Survey of the Italian Registry of Renal Biopsies. Frequency of the renal diseases for 7 consecutive years. Nephrol Dial Transplant 1997;12:418–26. 10.1093/ndt/12.3.418 9075118

[39] Zaza G, Bernich P, Lupo A. Incidence of primary glomerulonephritis in a large North-Eastern Italian area: a 13-year renal biopsy study. Nephrol Dial Transplant 2013;28:367–72. 10.1093/ndt/gfs437 23223218

[40] Micallef S, Balzan D, Bugeja M et al. P0823 Characterisation of histology proven glomerular diseases in the Maltese population: a national cross-sectional survey. Nephrol Dial Transplant 2020;35:iii1154. 10.1093/ndt/gfaa142.P0823

[41] Covic A, Vlad CE, Căruntu ID et al. Epidemiology of biopsy-proven glomerulonephritis in the past 25 years in the north-eastern area of Romania. Int Urol Nephrol 2022;54:365–76. 10.1007/s11255-021-02881-z 33991297

[42] Naumovic R, Pavlovic S, Stojkovic D et al. Renal biopsy registry from a single centre in Serbia: 20 years of experience. Nephrol Dial Transplant 2009;24:877–85. 10.1093/ndt/gfn564 18927123

[43] Stanković N, Vlahović P, Savić V. Histomorphological and clinical study of primary and secondary glomerulopathies in Southeast Serbia (20-year period of analysis). Vojnosanit Pregl 2013;70:1085–90. 10.2298/VSP110614027S 24450251

[44] Rivera F, Lopez-Gomez J, Perez-Garcia R. Frequency of renal pathology in Spain 1994-1999. Nephrol Dial Transplant 2002;17:1594–602. 10.1093/ndt/17.9.1594 12198210

[45] Peters B, Stegmayr B, Andersson Y et al. Increased risk of renal biopsy complications in patients with IgA-nephritis. Clin Exp Nephrol 2015;19:1135–41. 10.1007/s10157-015-1121-3 25951807

[46] Nanchen G, Schutzbach K, Rotman S et al. Incidence of glomerulonephritis in the western part of Switzerland over the last decade. Swiss Med Wkly 2020;150:w20353. 10.4414/smw.2020.20353 33085770

[47] McQuarrie E, Bell S, Campbell J et al. #5506 Native kidney biopsy: a national survey on, diagnoses and outcomes. Nephrol Dial Transplant 2023;38:i382–3.

[48] McQuarrie EP, Mackinnon B, McNeice V et al. The incidence of biopsy-proven IgA nephropathy is associated with multiple socioeconomic deprivation. Kidney Int 2014;85:198–203. 10.1038/ki.2013.329 24025641

[49] Heaf JG, Sørensen SS, Hansen A. Increased incidence and improved prognosis of glomerulonephritis: a national 30-year study. Clin Kidney J 2021;14:1594–602. 10.1093/ckj/sfaa169 34084455 PMC8162868

[50] Rehnberg J, Segelmark M, Ludvigsson JF et al. Validation of IgA nephropathy diagnosis in the Swedish Renal Registry. BMC Nephrol 2024;25:78. 10.1186/s12882-024-03512-2 PMC10910707 38438966

