# Supplementary material for: The prevalence of immunoglobulin A nephropathy in the European Union and the impact of the COVID-19 pandemic: an estimation approach utilizing the kidney biopsy frequency
Source: Clin Kidney J. 2025 Feb 28;18(4):sfaf068. doi: 10.1093/ckj/sfaf068 (PMC11997654; doi:10.1093/ckj/sfaf068)
Supplement: sfaf068_Supplemental_Files [file sfaf068_supplemental_files.zip › Supplement1_13Dec2024.docx]

**Supplement 1**

**S1.1: Literature search strategies**

Quality assurance: All literature reviews and subsequent data extraction involved two researchers to assure quality.

For the assessment of the COVID-19 pandemic impact on the kidney biopsy and IgAN frequency we performed a grey literature search to identify further relevant information, such as registry reports, in addition to search 3 and 4.

**Search 1: Incidence and prevalence of IgAN in Europe**

Database:

- EBM Reviews - Cochrane Central Register of Controlled Trials <February 2024>
- Embase <1974 to 2024 March 25>
- Ovid MEDLINE(R) Epub Ahead of Print and In-Process, In-Data-Review & Other Non-Indexed Citations <March 25, 2024>

Inclusion criteria:

- Population-based studies providing data on the incidence, prevalence, or risk of primary IgAN diagnosed by native kidney biopsy, or numbers to calculate those in the European general population (all ages or adults) which can be extrapolated to a defined region or country
- English language

Exclusion criteria:

- Articles not fulfilling the inclusion criteria.
- Recurrent IgAN in kidney transplant patients
- Studies on IgA vasculitis (IgAV)
- Low-quality studies as judged by epidemiologists, i.e. not appropriately defining the incidence/prevalent cases, study population, or containing a high risk of bias

Search terms:

| **#** | **Query** |
| --- | --- |
| 1 | exp "IgA glomerulonephritis"/ |
| 2 | (IGA or immunoglobulin A).mp. adj1 (exp nephropathy/ or nephropath*.mp. or exp glomerulopathy/ or glomerulopath*.mp. or exp Nephritis/ or Nephrit*.mp. or exp glomerulonephritis/ or glomerulonephrit*.mp.) |
| 3 | "berger* disease".mp. [mp=ti, ot, ab, fx, sh, hw, kw, tn, dm, mf, dv, kf, dq, bt, nm, ox, px, rx, ui, sy, ux, mx] |
| 4 | "Berger's Disease".mp. [mp=ti, ot, ab, fx, sh, hw, kw, tn, dm, mf, dv, kf, dq, bt, nm, ox, px, rx, ui, sy, ux, mx] |
| 5 | "IgAN".mp. [mp=ti, ot, ab, fx, sh, hw, kw, tn, dm, mf, dv, kf, dq, bt, nm, ox, px, rx, ui, sy, ux, mx] |
| 6 | "IgA glomerulonephritis".mp. [mp=ti, ot, ab, fx, sh, hw, kw, tn, dm, mf, dv, kf, dq, bt, nm, ox, px, rx, ui, sy, ux, mx] |
| 7 | "IgA nephropathy".mp. [mp=ti, ot, ab, fx, sh, hw, kw, tn, dm, mf, dv, kf, dq, bt, nm, ox, px, rx, ui, sy, ux, mx] |
| 8 | (IgA and glomerulopathy).mp. [mp=ti, ot, ab, fx, sh, hw, kw, tn, dm, mf, dv, kf, dq, bt, nm, ox, px, rx, ui, sy, ux, mx] |
| 9 | "Immunoglobulin A Nephropathy".mp. [mp=ti, ot, ab, fx, sh, hw, kw, tn, dm, mf, dv, kf, dq, bt, nm, ox, px, rx, ui, sy, ux, mx] |
| 10 | ("Immunoglobulin A" and Nephropathy).mp. [mp=ti, ot, ab, fx, sh, hw, kw, tn, dm, mf, dv, kf, dq, bt, nm, ox, px, rx, ui, sy, ux, mx] |
| 11 | ("Immunoglobulin A" and Glomerulonephritis).mp. [mp=ti, ot, ab, fx, sh, hw, kw, tn, dm, mf, dv, kf, dq, bt, nm, ox, px, rx, ui, sy, ux, mx] |
| 12 | "Nephritis, IGA".mp. [mp=ti, ot, ab, fx, sh, hw, kw, tn, dm, mf, dv, kf, dq, bt, nm, ox, px, rx, ui, sy, ux, mx] |
| 13 | "IgA Nephropathy 1".mp. [mp=ti, ot, ab, fx, sh, hw, kw, tn, dm, mf, dv, kf, dq, bt, nm, ox, px, rx, ui, sy, ux, mx] |
| 14 | "Glomerulonephritis, IGA".mp. [mp=ti, ot, ab, fx, sh, hw, kw, tn, dm, mf, dv, kf, dq, bt, nm, ox, px, rx, ui, sy, ux, mx] |
| 15 | 1 or 2 or 3 or 4 or 5 or 6 or 7 or 8 or 9 or 10 or 11 or 12 or 13 or 14 |
| 16 | (burden or incidence or incident or hazard or odds or risk or risks or ratio or frequency or frequencies or occur* or occurrence* or rate or rates or ratios or portion or number or magnitude or magnitudes or distribution or distributions or proportion* or percent* or prevalen* or epidemiolog* or morbidity or observational).mp. [mp=ti, ot, ab, fx, sh, hw, kw, tn, dm, mf, dv, kf, dq, bt, nm, ox, px, rx, ui, sy, ux, mx] |
| 17 | 15 and 16 |
| 18 | ("Case report" or "Case series").ti,ab. |
| 19 | animal experiment'/ or 'animal model'/ or 'practice guideline'/ or 'in vitro study'/ or placebo-controlled trial*/ or 'phase 1 clinical trial'/ or 'phase 2 clinical trial'/ or placebo-controlled trial*.ab,ti. |
| 20 | 18 or 19 |
| 21 | 17 not 20 |
| 22 | (Europ* or EU or Russi or Ukrain* or France or French or Spain or Spanish or Portug* or Sweden or Swedish or Norway or Norwegian or Finland or Finnish* or Scandinavi* or "Nordic countries" or Iceland* or Denmark or Danish or Faro* or German* or Poland or Polish or Ital* or "United Kingdom" or UK or Britain or British or Scotland or Scottish or Welsh or Wales or England or English or Ireland or Irish or Romani* or Belaru* or Kazakhstan or Greece or Greek or Bulgari* or Hungar* or Serbi* or Georgi* or Lithuan* or Latvi* or Croati* or Bosni* or Herzegovina or Slovaki* or Estoni* or Czech* or Moldov* or Albani* or Macedoni* or Turk* or Sloveni* or Montenegr* or Kosov* or Azerbaijan* or Armeni* or Cyprus or Cyprio* or Austri* or Switzerland or Swiss or Netherlands or Dutch or Holland or Belg* or Luxembourg* or Andorr* or Malta or Maltese or Liechtenstein or "San Marino" or Monaco or "Vatican City" or Baltic or Balka* or Slav* or Iberi* or Fennoscandian or Apennin* or Carpathian or Caucasus or Alpine or Caspian or "Black Sea" or Adriatic or Pannonian or Danubian or Dinaric or Macaronesia or "Channel islands" or Benelux or Jutland or Mediterranean or Thrace or Thrake).mp. [mp=ti, ot, ab, fx, sh, hw, kw, tn, dm, mf, dv, kf, dq, bt, nm, ox, px, rx, ui, sy, ux, mx] |
| 23 | 21 and 22 |
| 24 | remove duplicates from 23 |
| 25 | limit 24 to english language |
| 26 | limit 25 to yr="2022 -Current" |

**Search 2: Native kidney biopsy rate in Europe**

Database:

- Embase <1974 to 2024 March 27>
- Ovid MEDLINE(R) ALL <1946 to March 27, 2024>

Inclusion criteria:

- Population-based studies providing data on the annual native kidney biopsy rate, or numbers to calculate it in the European general population (all ages or adults) which can be extrapolated to a defined region or country
- English language

Exclusion criteria:

- Articles not fulfilling the inclusion criteria.
- Kidney biopsies in transplant patients
- Low-quality studies as judged by epidemiologists, i.e. not appropriately defining the incidence/prevalent cases, study population, or containing a high risk of bias

Search terms:

# Query

1 exp biopsy/

2 renal*.ab,kf,ti.

3 kidney*.ab,kf,ti.

4 2 or 3

5 (renal* or kidney*).ab,kf,ti. adj1 exp biopsy/

6 exp kidney biopsy/

7 kidney biops*.ab,kf,ti.

8 renal biops*.ab,kf,ti.

9 5 or 6 or 7 or 8

10 (Europ* or EU or Russi or Ukrain* or France or French or Spain or Spanish or Portug* or Sweden or Swedish or Norway or Norwegian or Finland or Finnish* or Scandinavi* or "Nordic countries" or Iceland* or Denmark or Danish or Faro* or German* or Poland or Polish or Ital* or "United Kingdom" or UK or Britain or British or Scotland or Scottish or Welsh or Wales or England or English or Ireland or Irish or Romani* or Belaru* or Kazakhstan or Greece or Greek or Bulgari* or Hungar* or Serbi* or Georgi* or Lithuan* or Latvi* or Croati* or Bosni* or Herzegovina or Slovaki* or Estoni* or Czech* or Moldov* or Albani* or Macedoni* or Turk* or Sloveni* or Montenegr* or Kosov* or Azerbaijan* or Armeni* or Cyprus or Cyprio* or Austri* or Switzerland or Swiss or Netherlands or Dutch or Holland or Belg* or Luxembourg* or Andorr* or Malta or Maltese or Liechtenstein or "San Marino" or Monaco or "Vatican City" or Baltic or Balka* or Slav* or Iberi* or Fennoscandian or Apennin* or Carpathian or Caucasus or Alpine or Caspian or "Black Sea" or Adriatic or Pannonian or Danubian or Dinaric or Macaronesia or "Channel islands" or Benelux or Jutland or Mediterranean or Thrace or Thrake).mp.

11 9 and 10

12 exp epidemiology/ or exp morbidity/

13 (burden or incidenc* or incident or frequenc* or occur* or occurrence* or rate or rates or proportion* or percent* or epidemiolog*).mp.

14 12 or 13

15 11 and 14

16 'animal experiment'/ or 'animal model'.mp. or 'nonhuman'/ or 'practice guideline'/

17 (case report* or case serie*).mp.

18 ('phase 1 clinical trial' or 'phase 2 clinical trial').mp.

19 'in vitro study'/

20 Randomized controlled trial*.mp.

21 Clinical trial*.mp.

22 Mouse model.mp.

23 placebo-controlled trial.mp.

24 rat model.mp.

25 mice model.mp.

26 16 or 17 or 18 or 19 or 20 or 21 or 22 or 23 or 24 or 25

27 15 not 26

28 limit 27 to english language

29 remove duplicates from 28

**Search 3: IgAN frequency during the COVID-19 pandemic**

Database:

- EBM Reviews - Cochrane Central Register of Controlled Trials <February 2024>
- Embase <1974 to 2024 March 25>
- Ovid MEDLINE(R) Epub Ahead of Print and In-Process, In-Data-Review & Other Non-Indexed Citations <March 25, 2024>

Inclusion criteria:

- Population-based studies from Europe reporting COVID-19 pandemic impact on the IgAN frequency, or the number of IgAN cases before, during, or after the pandemic
- Scientific articles (reviews, etc.) discussing the COVID-19 pandemic impact on the IgAN frequency
- English language

Exclusion criteria:

- Articles not fulfilling the inclusion criteria
- Articles including recurrent IgAN population only

Search terms:

| **#** | **Query** |
| --- | --- |
| 1 | exp "IgA glomerulonephritis"/ |
| 2 | (IGA or immunoglobulin A).mp. adj1 (exp nephropathy/ or nephropath*.mp. or exp glomerulopathy/ or glomerulopath*.mp. or exp Nephritis/ or Nephrit*.mp. or exp glomerulonephritis/ or glomerulonephrit*.mp.) |
| 3 | "berger* disease".mp. [mp=ti, ot, ab, fx, sh, hw, kw, tn, dm, mf, dv, kf, dq, bt, nm, ox, px, rx, ui, sy, ux, mx] |
| 4 | "Berger's Disease".mp. [mp=ti, ot, ab, fx, sh, hw, kw, tn, dm, mf, dv, kf, dq, bt, nm, ox, px, rx, ui, sy, ux, mx] |
| 5 | "IgAN".mp. [mp=ti, ot, ab, fx, sh, hw, kw, tn, dm, mf, dv, kf, dq, bt, nm, ox, px, rx, ui, sy, ux, mx] |
| 6 | "IgA glomerulonephritis".mp. [mp=ti, ot, ab, fx, sh, hw, kw, tn, dm, mf, dv, kf, dq, bt, nm, ox, px, rx, ui, sy, ux, mx] |
| 7 | "IgA nephropathy".mp. [mp=ti, ot, ab, fx, sh, hw, kw, tn, dm, mf, dv, kf, dq, bt, nm, ox, px, rx, ui, sy, ux, mx] |
| 8 | (IgA and glomerulopathy).mp. [mp=ti, ot, ab, fx, sh, hw, kw, tn, dm, mf, dv, kf, dq, bt, nm, ox, px, rx, ui, sy, ux, mx] |
| 9 | "Immunoglobulin A Nephropathy".mp. [mp=ti, ot, ab, fx, sh, hw, kw, tn, dm, mf, dv, kf, dq, bt, nm, ox, px, rx, ui, sy, ux, mx] |
| 10 | ("Immunoglobulin A" and Nephropathy).mp. [mp=ti, ot, ab, fx, sh, hw, kw, tn, dm, mf, dv, kf, dq, bt, nm, ox, px, rx, ui, sy, ux, mx] |
| 11 | ("Immunoglobulin A" and Glomerulonephritis).mp. [mp=ti, ot, ab, fx, sh, hw, kw, tn, dm, mf, dv, kf, dq, bt, nm, ox, px, rx, ui, sy, ux, mx] |
| 12 | "Nephritis, IGA".mp. [mp=ti, ot, ab, fx, sh, hw, kw, tn, dm, mf, dv, kf, dq, bt, nm, ox, px, rx, ui, sy, ux, mx] |
| 13 | "IgA Nephropathy 1".mp. [mp=ti, ot, ab, fx, sh, hw, kw, tn, dm, mf, dv, kf, dq, bt, nm, ox, px, rx, ui, sy, ux, mx] |
| 14 | "Glomerulonephritis, IGA".mp. [mp=ti, ot, ab, fx, sh, hw, kw, tn, dm, mf, dv, kf, dq, bt, nm, ox, px, rx, ui, sy, ux, mx] |
| 15 | 1 or 2 or 3 or 4 or 5 or 6 or 7 or 8 or 9 or 10 or 11 or 12 or 13 or 14 |
| 16 | (burden or incidence or incident or hazard or odds or risk or risks or ratio or frequency or frequencies or occur* or occurrence* or rate or rates or ratios or portion or number or magnitude or magnitudes or distribution or distributions or proportion* or percent* or prevalen* or epidemiolog* or morbidity or observational).mp. [mp=ti, ot, ab, fx, sh, hw, kw, tn, dm, mf, dv, kf, dq, bt, nm, ox, px, rx, ui, sy, ux, mx] |
| 17 | 15 and 16 |
| 18 | ("Case report" or "Case series").ti,ab. |
| 19 | animal experiment'/ or 'animal model'/ or 'practice guideline'/ or 'in vitro study'/ or placebo-controlled trial*/ or 'phase 1 clinical trial'/ or 'phase 2 clinical trial'/ or placebo-controlled trial*.ab,ti. |
| 20 | 18 or 19 |
| 21 | 17 not 20 |
| 22 | (Europ* or EU or Russi or Ukrain* or France or French or Spain or Spanish or Portug* or Sweden or Swedish or Norway or Norwegian or Finland or Finnish* or Scandinavi* or "Nordic countries" or Iceland* or Denmark or Danish or Faro* or German* or Poland or Polish or Ital* or "United Kingdom" or UK or Britain or British or Scotland or Scottish or Welsh or Wales or England or English or Ireland or Irish or Romani* or Belaru* or Kazakhstan or Greece or Greek or Bulgari* or Hungar* or Serbi* or Georgi* or Lithuan* or Latvi* or Croati* or Bosni* or Herzegovina or Slovaki* or Estoni* or Czech* or Moldov* or Albani* or Macedoni* or Turk* or Sloveni* or Montenegr* or Kosov* or Azerbaijan* or Armeni* or Cyprus or Cyprio* or Austri* or Switzerland or Swiss or Netherlands or Dutch or Holland or Belg* or Luxembourg* or Andorr* or Malta or Maltese or Liechtenstein or "San Marino" or Monaco or "Vatican City" or Baltic or Balka* or Slav* or Iberi* or Fennoscandian or Apennin* or Carpathian or Caucasus or Alpine or Caspian or "Black Sea" or Adriatic or Pannonian or Danubian or Dinaric or Macaronesia or "Channel islands" or Benelux or Jutland or Mediterranean or Thrace or Thrake).mp. [mp=ti, ot, ab, fx, sh, hw, kw, tn, dm, mf, dv, kf, dq, bt, nm, ox, px, rx, ui, sy, ux, mx] |
| 23 | 21 and 22 |
| 24 | remove duplicates from 23 |
| 25 | limit 24 to english language |
| 26 | limit 25 to covid-19 [Limit not valid in CCTR; records were retained] |

**Search 4: Native kidney biopsy rate during the COVID-19 pandemic**

Database:

- Embase <1974 to 2024 March 27>
- Ovid MEDLINE(R) ALL <1946 to March 27, 2024>

Inclusion criteria:

- Population-based studies from Europe reporting COVID-19 pandemic impact on the native kidney biopsy rate, or the number of native kidney biopsies before, during, or after the pandemic
- Scientific articles (reviews, etc.) discussing the COVID-19 pandemic impact on the native kidney biopsy rate
- English language

Exclusion criteria:

- Articles not fulfilling the inclusion criteria
- Articles including kidney transplant population only

Search terms:

# Query

1 exp biopsy/

2 renal*.ab,kf,ti.

3 kidney*.ab,kf,ti.

4 2 or 3

5 (renal* or kidney*).ab,kf,ti. adj1 exp biopsy/

6 exp kidney biopsy/

7 kidney biops*.ab,kf,ti.

8 renal biops*.ab,kf,ti.

9 5 or 6 or 7 or 8

10 exp Covid-19/

11 ("Coronavirus disease-19" or "Coronavirus disease19" or "coronavirus disease 2019" or "2019-nCoV" or "2019 nCoV").ab,kf,ti.

12 ("SARS-CoV-2" or "SARS CoV-2" or "SARS-COV2" or "SARS COV 2" or "SARS-COV-2").ab,kf,ti.

13 (COVID-19 or COVID*3 or "Coronavirus-2" or "Coronavirus 2" or "SARS Coronavirus 2").ab,kf,ti.

14 10 or 11 or 12 or 13

15 9 and 14

16 (Europ* or EU or Russi or Ukrain* or France or French or Spain or Spanish or Portug* or Sweden or Swedish or Norway or Norwegian or Finland or Finnish* or Scandinavi* or "Nordic countries" or Iceland* or Denmark or Danish or Faro* or German* or Poland or Polish or Ital* or "United Kingdom" or UK or Britain or British or Scotland or Scottish or Welsh or Wales or England or English or Ireland or Irish or Romani* or Belaru* or Kazakhstan or Greece or Greek or Bulgari* or Hungar* or Serbi* or Georgi* or Lithuan* or Latvi* or Croati* or Bosni* or Herzegovina or Slovaki* or Estoni* or Czech* or Moldov* or Albani* or Macedoni* or Turk* or Sloveni* or Montenegr* or Kosov* or Azerbaijan* or Armeni* or Cyprus or Cyprio* or Austri* or Switzerland or Swiss or Netherlands or Dutch or Holland or Belg* or Luxembourg* or Andorr* or Malta or Maltese or Liechtenstein or "San Marino" or Monaco or "Vatican City" or Baltic or Balka* or Slav* or Iberi* or Fennoscandian or Apennin* or Carpathian or Caucasus or Alpine or Caspian or "Black Sea" or Adriatic or Pannonian or Danubian or Dinaric or Macaronesia or "Channel islands" or Benelux or Jutland or Mediterranean or Thrace or Thrake).mp.

17 15 and 16

18 exp epidemiology/ or exp morbidity/

19 (burden or incidenc* or incident or frequenc* or occur* or occurrence* or rate or rates or proportion* or percent* or epidemiolog*).mp.

20 18 or 19

21 17 and 20

22 'animal experiment'/ or 'animal model'.mp. or 'nonhuman'/ or 'practice guideline'/

23 (case report* or case serie*).mp.

24 ('phase 1 clinical trial' or 'phase 2 clinical trial').mp.

25 'in vitro study'/

26 Randomized controlled trial*.mp.

27 Clinical trial*.mp.

28 Mouse model.mp.

29 placebo-controlled trial.mp.

30 rat model.mp.

31 mice model.mp.

32 22 or 23 or 24 or 25 or 26 or 27 or 28 or 29 or 30 or 31

33 21 not 32

34 limit 33 to english language

35 remove duplicates from 34

**S1.2: Detailed methods of country-specific prevalence estimation**

**Quality assurance:**

To ensure quality of the results, all data extraction steps were done by two researchers and double programming was done for all analyses.

**Prevalence estimation:**

The country-specific IgAN prevalence estimates across EU countries were reported or calculated based on the following scenarios:

*Scenario 1: Publication reported the country-specific IgAN prevalence.*

The prevalence estimate was used as reported if it was of acceptable quality, i.e. based on a well-defined population-based study. If several high-quality prevalence estimates were available for one country, the most recent estimate was used.

*Scenario 2: No country-specific IgAN prevalence estimate available, but country-specific IgAN annual incidence was available*

The country-specific prevalence was reported using the following formula:

$${Prevalence}_{country}= {Incidence}_{country}*Disease duration$$

If several annual incidence estimates were available for one country, the most recent and appropriate estimate was used.

The disease duration was based on a recent study from Sweden (*Jarrick et al. (2019) Mortality in IgA Nephropathy: A Nationwide Population-Based Cohort Study. JASN; 30: 866-876*): Study results suggested that 50% of patients developed kidney failure within 30 years after diagnosis with the highest risk for progressing to kidney failure in the first year. Kidney failure was assumed to be the end of primary IgAN diagnosed by native kidney biopsy because it is the end of kidney survival and recurrent IgAN in transplanted kidney was out of scope from this analysis. Therefore, the IgAN prevalence was calculated using 30 years as disease duration. Assumptions around the disease duration were tested in a sensitivity analysis (see Supplement 3, Table S3.2)

*Scenario 3: No country-specific IgAN prevalence or incidence available, but country-specific biopsy rate available.*

The annual IgAN incidence was estimated through a linear regression between the annual kidney biopsy rate and annual IgAN incidence using all available data from European countries and regions, not limited to EU, with sufficient data, i.e. IgAN incidence and biopsy rate available or calculatable, to increase the robustness of the linear regression. The annual biopsy rate was the independent and the annual IgAN incidence the dependent variable. The linear regression model was fitted without an intercept to force the regression line through the origin. Consequently, the incidence was estimated by multiplying the biopsy rate with the slope.

Several estimates from the same country or region may have been used for the linear regression if available. However, in case of duplicate estimates, only the most recent estimate was included. Duplicate estimates were defined as estimates from the same population and during the same or overlapping time period.

If several biopsy rate estimates were available for one country, the most recent appropriate estimate was used for the estimation of the respective IgAN incidence through the linear regression.

After estimating the IgAN annual incidence through linear regression, the IgAN prevalence was calculated as described in Scenario 2.

*Scenario 4: No relevant country-specific information available.*

To identify the most appropriate biopsy rate for a country with no published information, the Healthcare access and quality index (HAQI) for the most recent available year, i.e. 2022, from European countries (not limited to EU) as reported in the Global Burden of Disease (GBD) study was utilized (*Global Burden of Disease (GBD) data, published by the Institute for Health Metrics and Evaluation (IHME), were queried via the Data Explorer. Accessed on 2024-11-18*). For details of the HAQI derivation see [GBD 2019 Healthcare Access and Quality Collaborators](https://www.thelancet.com/journals/langlo/article/PIIS2214-109X(22)00429-6/fulltext) (DOI: [10.1016/S2214-109X(22)00429-6](https://doi.org/10.1016/S2214-109X(22)00429-6)). For this analysis, the HAQI is transformed into four categories based on the range of HAQI in the European countries:

- Low HAQI: [${HAQI}_{min}$ , ${HAQI}_{min}$+(${HAQI}_{max}$ -${HAQI}_{min}$)/4)
- Medium-low HAQI: [${HAQI}_{min}$+(${HAQI}_{max}$ −${HAQI}_{min}$)/4 , ${HAQI}_{min}$+2∗(${HAQI}_{max}$ −${HAQI}_{min}$)/4)
- Medium-high HAQI: [${HAQI}_{min}$+2∗(${HAQI}_{max}$ −${HAQI}_{min}$)/4 , ${HAQI}_{min}$+3∗(${HAQI}_{max}$ −${HAQI}_{min}$)/4)
- High HAQI: [${HAQI}_{min}$+3∗(${HAQI}_{max}$ −${HAQI}_{min}$)/4 , ${HAQI}_{max}$]

with ${HAQI}_{min}$ = lowest HAQI and ${HAQI}_{max}$ = highest HAQI.

The median biopsy rate of all available biopsy rates from countries within the same HAQI category which have a shared border with the respective country was assumed as the biopsy rate. If there were no neighbors with available data within the same HAQI category, the median biopsy rate of all data from countries within the same HAQI category was assumed.

The country-specific prevalence was calculated as described in Scenario 3.
