# Supplementary material for: The prevalence of immunoglobulin A nephropathy in the European Union and the impact of the COVID-19 pandemic: an estimation approach utilizing the kidney biopsy frequency
Source: Clin Kidney J. 2025 Feb 28;18(4):sfaf068. doi: 10.1093/ckj/sfaf068 (PMC11997654; doi:10.1093/ckj/sfaf068)
Supplement: sfaf068_Supplemental_Files [file sfaf068_supplemental_files.zip › Supplement3_13Dec2024.docx]

**Supplement 3**


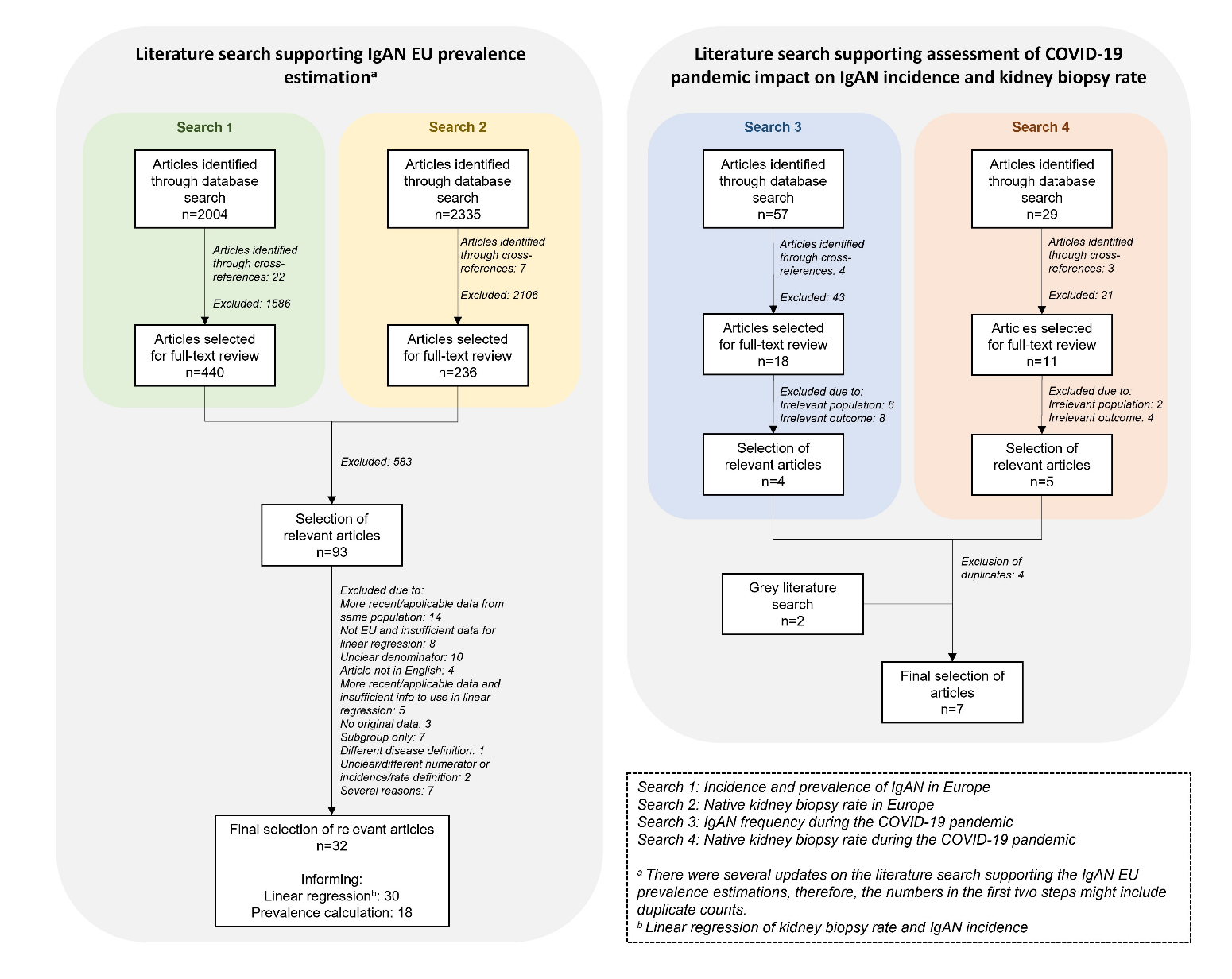


**Figure S3.1:** PRISMA flow diagram of the literature reviews.


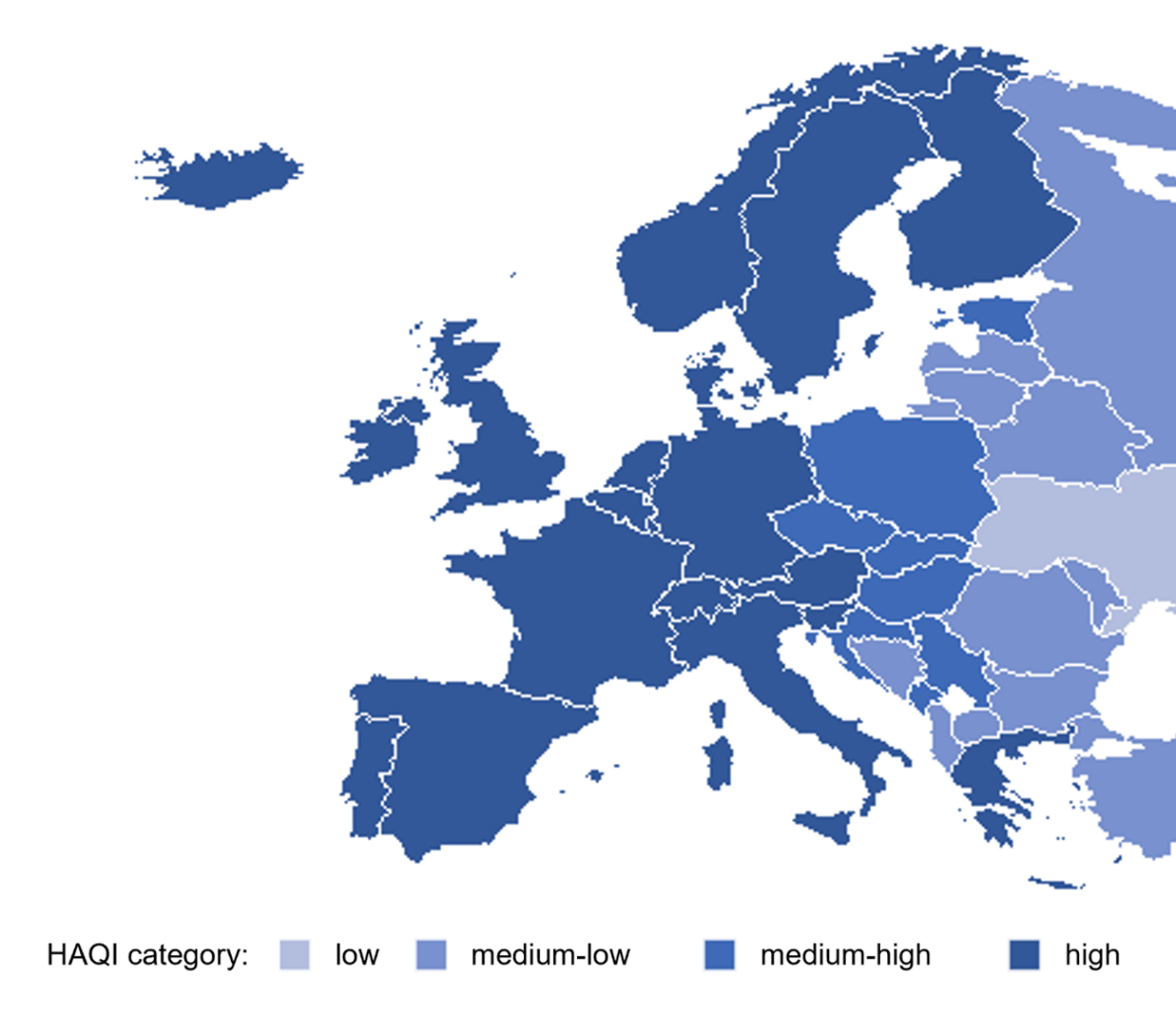


**Figure S3.2** Healthcare Access and Quality Index (HAQI) categories defined based on Global Burden of Disease (GBD) study in Europe (*Global Burden of Disease (GBD) data, published by the Institute for Health Metrics and Evaluation (IHME), were queried via the Data Explorer. Accessed on 2024-08-13*).


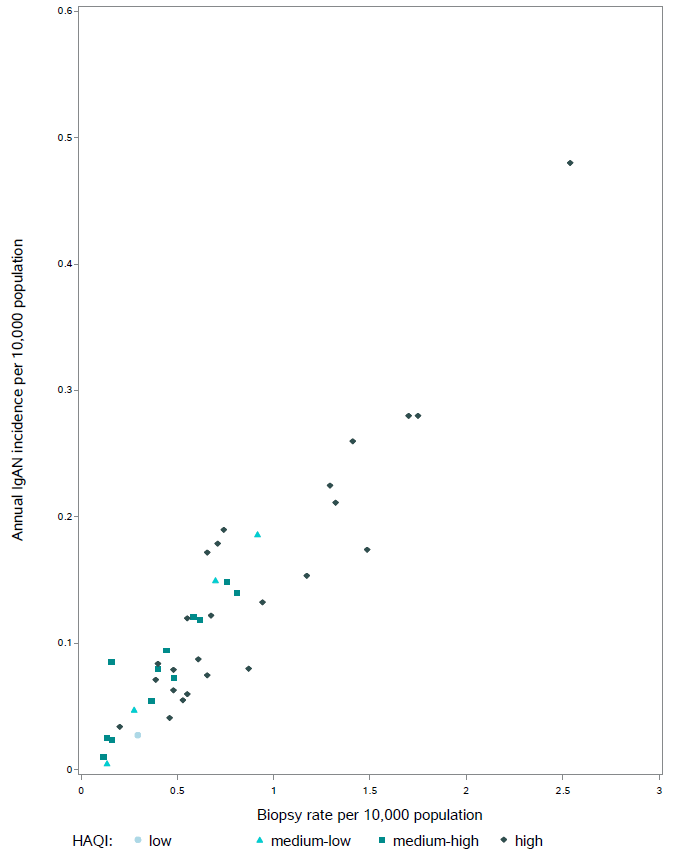


**Figure S3.3** Linear relationship of annual IgAN incidence and annual native kidney biopsy rate by Healthcare Access and Quality Index (HAQI) category based on Global Burden of Disease (GBD) data (*Global Burden of Disease (GBD) data, published by the Institute for Health Metrics and Evaluation (IHME), were queried via the Data Explorer. Accessed on 2024-11-18*).

**Table S3.1** Assumed annual native kidney biopsy rates and HAQI for countries without published information on biopsy rate or IgAN incidence.

| Country | HAQI category^a^ | Assumed biopsy rate per 10 000 population^b^ |
| --- | --- | --- |
| Austria | high | 0.55 |
| Bulgaria | medium-low | 0.14 |
| Greece | high | 0.67 |
| Ireland | high | 0.71 |
| Latvia | medium-low | 0.70 |
| Luxembourg | high | 1.29 |
| Portugal | high | 0.48 |
| Slovakia | medium-high | 0.46 |
| Slovenia | high | 0.54 |
| ^a^ Categories created based on GBD HAQI (*Global Burden of Disease (GBD) data, published by the Institute for Health Metrics and Evaluation (IHME), were queried via the Data Explorer. Accessed on 2024-11-18*): low: 58.31-67.28; medium-low: 67.29-76.27; medium-high: 76.28-85.26; high: 85.27-94.26.  ^b^ Assumed as median biopsy rate of all available biopsy rates from European neighbor countries within same HAQI category (Austria, Bulgaria, Ireland, Latvia, Luxembourg, Portugal, Slovakia, Slovenia), or if no neighbors with available data, median biopsy rate of all European countries within same HAQI category (Greece).  Abbreviations: HAQI=Healthcare Access and Quality Index. | | |

**Table S3.2:** Sensitivity analyses to test assumptions around disease duration and assumed biopsy rates for countries without relevant information

| Analysis | Disease duration (years) | Assumed biopsy rate for countries w/o info per 10 000 | Calculated prevalence in EU per 10 000 |
| --- | --- | --- | --- |
| Primary analysis | 30^a^ | 0.14-1.29 | 4.31 |
| Sensitivity analysis 1 | **11.4^b^** | 0.14-1.29 | 1.64 |
| Sensitivity analysis 2 | **37.4^c^** | 0.14-1.29 | 5.37 |
| Sensitivity analysis 3 | 30^a^ | **0.61**  **(median of all biopsy rates after 2010)** | 4.36 |
| Sensitivity analysis 4 | 30^a^ | **0.15-0.75**  **(median of all neighboring countries regardless of HAQI^d^)** | 4.29 |
| Bold text indicates the changed assumption for each sensitivity analysis.  ^a^ Median time from IgAN diagnosis to kidney failure among Swedish IgAN patients (*Jarrick et al. (2019) Mortality in IgA Nephropathy: A Nationwide Population-Based Cohort Study. JASN; 30: 866-876*).  ^b^ Median kidney survival based on UK National Registry of Rare Kidney Diseases (RaDaR) (*Pitcher et al (2023) Long-Term Outcomes in IgA Nephropathy. CJASN; 18: 727-738*).  ^c^ Calculated by subtracting the median age at diagnosis (39.6 years) from the median age at death (77.0 years) among Swedish IgAN patients (*Jarrick et al. (2019) Mortality in IgA Nephropathy: A Nationwide Population-Based Cohort Study. JASN; 30: 866-876*).  ^d^ For Greece, there were no neighbor countries with an available biopsy rate, therefore, as an exception, European countries sharing a sea border with Greece (i.e. Italy, Malta, Cyprus) were also assumed as neighbor countries. | | | |
